# The Medical Student and Dental Training

**Published:** 1909-04-17

**Authors:** 


### Dental Department.

### THE MEDICAL STUDENT AND DENTAL TRAINING.

(*From a Correspondent.*)

Remembering the great increase in the number of subjects included in the curriculum of a general medical education at the present day, and the great demand made on the time and labour of medical students, one naturally hesitates to suggest any widening of their field of activities. It is only because there is a serious omission, a very necessary piece of apparatus wanting, in an otherwise fairly complete training for the relief of human suffering, that we venture to suggest a further slight extension of an already formidable syllabus. We refer to the treatment of disease and pain arising from some defect in the dental mechanism.

We are mindful of the fact that there exists a separate profession, a body of men trained specially for the treatment of such conditions; but we maintain that a medical man should be master of the situation when consulted on any question of pain and disease; he should be able to diagnose the condition and take the recognised means for the relief of the pain. At the present time the only training a medical student gets, as far as the treatment of diseased teeth is concerned, is an optional month spent in the extraction-room. Now the extraction of teeth is not the recognised method of treatment of dental disease at the present day, except under exceptional circumstances; and while the dental profession is straining every endeavour to preserve both the temporary and permanent masticatory apparatus intact, the medical man, when necessity compels him to act, is driven by want of knowledge to the obsolete and deplorable practice of extracting.

We know that it is only in exceptional cases that necessity compels a medical man to take action where teeth are concerned. In towns and their immediate vicinity special dental skill is almost always available, and the medical practitioner rightly refers his patient to a dentist. Now by reason of the fact that a dentist confines himself to the treatment of conditions arising from one small portion of the human frame, while the medical man treats the whole, there are far more medical than dental practitioners; and while dental practitioners are mostly confined to the large centres of the population, the medical man may often find himself far removed from those centres, in a small town or village, on board ship, or in an out-of-the-way place in the colonies and elsewhere abroad. Under these circumstances a doctor may be consulted by a patient in the throes of most acute, unbearable pain, when action is imperative, and here it is that he feels he has a vulnerable point in his armour; and he is driven, reluctantly no doubt, for very few medical men feel at home with a pair of forceps, to the extraction of the faulty tooth.

There are other reasons, too, why medical men should have some knowledge of dental practice; they are always running against dental disease, in defects in the alimentary system, in blood diseases, and many other conditions. Their first thought is to examine the mouth, the gateway of the system, and the throat, for here abnormal conditions may be seen directly with the eye, always the easiest and most satisfactory means of diagnosis, and in the mouth the teeth require their most careful consideration. It is the practice now to eliminate this factor as a possible source of the ailment by recommending dental treatment. It is here that some acquaintance with what is and what is not good dental practice is useful, not that the medical man is called upon to suggest what particular treatment is to be carried out, but rather that he should refrain from insisting on treatment which is wrong. How often have we been consulted by patients who have told us that their doctor has insisted that they must have all their teeth out, and on examining the mouth we have found that though their teeth are apparently in a deplorable condition, yet really they are well within the scope of conservative dentistry. It is difficult to persuade such patients that by appropriate treatment their teeth may be saved, and from being a possible cause of their more general ill-health, may be made an important factor in their ultimate recovery.

Under the Act for the medical inspection of school children medical men are called upon to make a very thorough examination and report upon the general physical condition of the children, and such report necessitates a statement as to the condition of the teeth. Practice in the examination of mouths, and a knowledge of the conditions appertaining to the shedding of the temporary and eruption of the permanent dentition, are a necessity for the correct rendering of such a report.

Again, the family doctor occupies with some people a unique position; he is a sort of father confessor, an adviser on all matters relating to their general well-being. He may be asked to express an opinion on the advisability of the work Mr. So-and-So wishes to do for them, or he may be asked his opinion on the work that has been done. He is frequently asked to recommend a dentist, and if he has some knowledge of what is best in modern dentistry, and is able to appreciate good work when he sees it (and he often gets an opportunity of looking at dental work), so much the better advice can he give his patient.

We are far from advising anything like an elaborate training in dentistry for the medical student, but we do think he should be taught how to deal with a case of acute odontalgia otherwise than by extraction. In some hospitals the dental student holds an appointment for a month, called "Casualty Dressing"; here he deals with those cases where the patient comes up complaining of acute toothache, where the only treatment contemplated is the treatment of the one tooth causing trouble. He has under treatment such conditions as hyperæmia of the pulp, acute pulpitis, suppurative pulpitis, and periodontitis. The immediate relief of the symptoms in these conditions is simple, and the experience gained is invaluable. We think all medical students, especially if they contemplate practising in remote places, would find the experience gained by holding such an appointment well worth the extra time and trouble. The outfit necessary for such treatment need not be excessive, as all that is required is to relieve the pain and the adaptation of the cavity in the tooth to the secure retention of the temporary filling which is to hold the medicament used where it is required. Whilst holding this appointment a series of six or eight lectures given by the dental surgeon attached to the hospital might be arranged; they might comprise a brief description of the anatomy of a tooth, a description of the temporary and permanent dentition, the theory as to the causes of dental disease, the value of early conservative treatment of decayed teeth, some remarks on diet and its effect on teeth, a general outline of the various means employed by dentists in the restoration of a defective masticatory apparatus, giving the general opinion of the dental profession on the value of fillings and filling materials, crown and bridge work, when it is indicated, and when not, the value of orthodontia in regard to children's teeth, and other matters relating to the care of the teeth. After holding such an appointment a medical man would be in a position to deal with any urgent case of toothache, and he would feel at home when asked his advice on any question relating to the teeth and the practice of dentistry.

